# Non-negligible greenhouse gases from urban sewer system

**DOI:** 10.1186/s13068-019-1441-8

**Published:** 2019-04-27

**Authors:** Pengkang Jin, Yonggang Gu, Xuan Shi, Wenna Yang

**Affiliations:** School of Environmental and Municipal Engineering, Xi’an University of Architecture and Technology, Xi’an, 710055 Shaanxi China

**Keywords:** Urban sewer system, Greenhouse gases, Contaminant degradation, Pollutant transformation, Annotated pathway, Global warming

## Abstract

**Background:**

The urban sewer system is an important component of urban water infrastructure for sewage collection and transportation, and in-sewer transportation of sewage can cause multitudinous contaminant degradations which lead to formation of gaseous products. Although the greenhouse gases of methane and carbon dioxide have been confirmed to consist in the gaseous products, the mechanisms of greenhouse gas generation were unclear and the significances of greenhouse gases emission from sewers were generally underestimated.

**Results:**

In this study, 3 years of monitoring was conducted to evaluate the greenhouse gases emission in 37-km-long urban sewer systems covering 13 km^2^. The results showed that the emission of carbon dioxide and methane was extensively existing in sewers, and especially, exhibited a characteristic of regional difference. In order to reveal the formation mechanism of carbon dioxide and methane in sewers, the metagenomic approach was utilized to analyze the annotated pathways and homologous bio-enzymes, and it indicated that fourteen pivotal annotated pathways were involved in the carbon dioxide and methane generation. According to the metagenomics and 3-year monitoring results, the total amounts of carbon dioxide and methane emission in sewers were calculated by the transformation venation of contaminants (such as methyl alcohol, methylamine and acetic acid along branch sewer, sub-main sewer and main sewer, respectively). The calculation results showed that the total greenhouse gas emissions in sewer were calculated to be 199 t/day in Xi’an, and if scaling up as population proportion, the greenhouse gas emission from sewer systems in China could be 30,685 t/day. Comparing with the greenhouse gas emissions from different metropolises (New York City, London and Tokyo) and industries (dairy farms, automobile production and steel enterprises), the amount of greenhouse gases produced by the urban sewer system is much higher.

**Conclusions:**

This study revealed the transformation pathways of contaminants which promoted the generation of greenhouse gases in sewers. Based on this analysis, the greenhouse gas emissions along sewer systems were calculated. The results indicate that the greenhouse gas emission from sewer systems is non-negligible, and should be attracted sufficient attention.

**Electronic supplementary material:**

The online version of this article (10.1186/s13068-019-1441-8) contains supplementary material, which is available to authorized users.

## Introduction

Due to the large and increasing amount of greenhouse gases, global warming has become an indisputable scientific fact, and a hot research topic worldwide [1]. In 2009, the United Nations Conference was held in Copenhagen, Denmark, to discuss the effect of climate change [2]. Therefore, how to effectively reduce carbon emissions has become one of the focuses of international political, economic, and academic research.

In general, industrial engineering, agriculture, and animal husbandry are considered the most important sources of greenhouse gas emissions in the world. However, during the wastewater treatment process, methane generation and incineration can also lead the emissions of large amount of greenhouse gases [3]. Therefore, the significance of greenhouse gas emission from wastewater treatment plants has been increasingly acknowledged in recent years, especially during the sewage sludge treatment process. To solve this environmental problem, two authoritative global framework standard and mathematical models [Intergovernmental Panel on Climate Change (IPCC) and Clean Development Mechanism (CDM)] [4–6] were proposed to provide a basis for reducing greenhouse gas emissions during sewage sludge treatment. As the front line of the wastewater treatment process, urban sewer systems transport a large amount of sewage every day. The sewage in sewers contains abundant contaminants, particularly the organic matter. The energetic metabolism of the microbial communities in biofilms attached to pipes and the anaerobic environment in sewer sediments provide suitable conditions for the transformation of contaminants. Due to these diverse bioreactions, especially fermentation, as verified in our previous studies [7, 8], significant amounts of greenhouse gases such as methane and carbon dioxide can be generated in sewers. Sewer systems have been constructed in most regions of the world, and the flow time of sewage in sewers is generally more than 8–10 h [9]. In addition, the length of a sewer pipe is longer than 10 km in China [10], which can almost guarantee sufficient conditions for greenhouse gas generation in sewers. The excessive emission of greenhouse gases can induce climate change, and influence the economic and agricultural development of countries, which will, in turn, affect the countries’ development strategies. Therefore, evaluating the sources of greenhouse gas emission is of great importance. Due to the obvious generation potential of sewers, the emission of greenhouse gases should be given more attention. However, to the best of our knowledge, the greenhouse gas generation pathways in sewers have not been comprehensively determined.

During the degradation process of diverse contaminants, carbon dioxide and methane can be generated in sewer [11, 12]. Therefore, the distribution characteristics of contaminants in a sewer affect the emissions of carbon dioxide and methane [13]. Additionally, the functions of different sewer systems can affect this process [14, 15]. Thus, in this study, four kinds of typical sewer system segments covering an area of 13 km^2^ and including different functional areas in Xi’an were chosen to evaluate the emission of greenhouse gases (methane and carbon dioxide). Through the metagenome analysis, the generation pathways of methane and carbon dioxide were revealed, and the variations of substrates (carbohydrates, proteins, and lipids) were determined. Based on the results, the emission values of methane and carbon dioxide in urban sewer systems were calculated, and the effects of these greenhouse gases from sewer systems were evaluated. Thus, this study provides a new understanding of the urban sewer systems, and serve as a basis for paying more attention to greenhouse gas emissions from urban sewer systems around the world.

## Results and discussion

### Investigation of greenhouse gas emission from urban sewer systems in Xi’an

In general, there are four kinds of dominant functional areas in cities: residential areas, comprehensive service areas, schools, and business districts. Due to the diverse functions of these areas, the types of wastewater in these areas differ (such as toilet, kitchen, wash wastewater, and rain water) and the amounts of these types of wastewater change markedly under different weather conditions (as shown in Additional file 1: Figure S1). The wastewater flows from the outfall to branch sewer pipe, then to the sub-main sewer pipe, and finally to the main sewer pipe. Due to the different flow conditions in the three ranks of sewer, the transformation of diverse contaminants in different types of wastewater might change, which could further influence the characteristics of greenhouse gas generation.

**Fig. 1:**
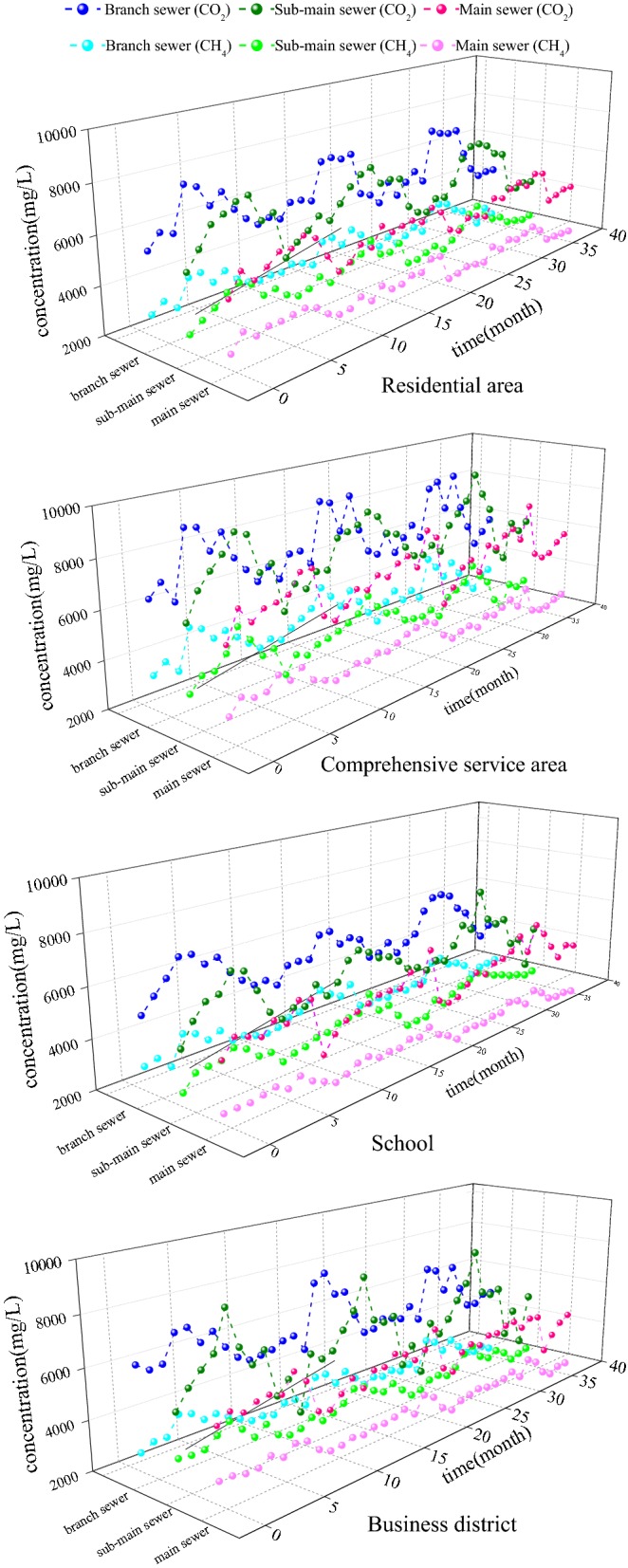
Greenhouse gas emission from sewers in residential areas, comprehensive service areas, schools, and business districts

To verify this hypothesis, the concentrations of CO_2_ and CH_4_ were monitored along the sewer under different conditions. As shown in Fig. 1, the production of greenhouse gases was highest in branch pipes, followed by the sub-main pipes and main pipes. Furthermore, the generation rates of greenhouse gases in separate sewer systems were higher than those in combined sewer systems. Based on the statistical results, the average concentrations of CO_2_ in branch pipe, sub-main pipe, and main pipe were 5916 mg/L, 2871 mg/L, and 1830 mg/L, respectively, and the average CH_4_ concentrations were 2937 mg/L, 1445 mg/L and 914 mg/L, respectively. In Xi’an, the lengths of branch pipes, sub-main pipes, and main pipes are 247,521 m, 219,506 m, and 371,873 m, respectively. The total amounts of CO_2_ and CH_4_ can then be calculated (15 t/day and 8 t/day, respectively). Because the influence factors of CO_2_ and CH_4_ as greenhouse gases are 1 and 23 [16], respectively, the total greenhouse gas amount in Xi’an can be calculated (188 t/day). The results indicated that the emission of greenhouse gases from urban sewer systems cannot be neglected. Due to the complex condition in real urban sewers, investigations of the greenhouse gas emission values should be further verified by analyzing the biological generation pathways (i.e., the degradation of organic matter).

### Analysis of greenhouse gas generation pathway

**Fig. 2:**
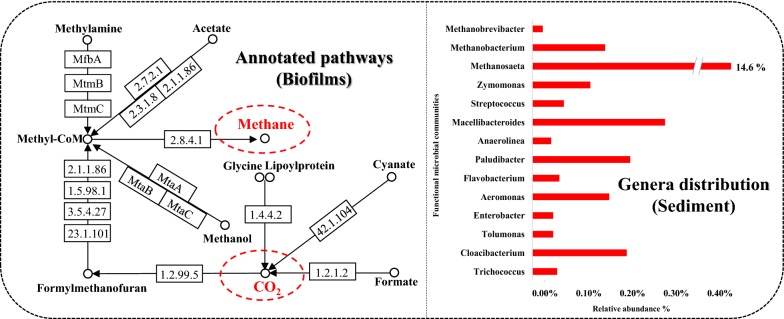
The annotated pathway for greenhouse gas production in urban sewer system

To comprehensively understand the greenhouse gas generation pathways in sewer, a metagenome analysis was used in this study. Kyoto Encyclopedia of Genes and Genomes Pathway Database (KEGG) analysis identified key genes implicated in the methane and carbon pathways (as shown in Fig. 2). The results showed that various enzymes encoded by corresponding functional genes took part in CH_4_ and CO_2_ production in urban sewer system. Within the methane pathway, the genes coding for Methylcoenzyme M (Methyl-CoM; EC 2.8.4.1) had the highest abundance in the biofilm sample. In addition, methylamine, methanol, formate, and acetate utilized diverse enzymes, including EC 2.7.2.1, EC 23.1.101, and EC 1.2.99.5, to generate the Methyl-CoM and then promote the accumulation of methane in urban sewer system. The annotated formate dehydrogenase (EC 1.2.1.2) and glycine dehydrogenase (EC 1.4.4.2) played an important role in the CO_2_ producing pathway, and the relative abundances of microbial communities that decomposed carbohydrates, proteins, and lipids to CO_2_ were non-negligible in the sewer system. Furthermore, methanogens and fermentative bacteria were also relatively abundant in the sewer sediment. Therefore, CH_4_ and CO_2_ can easily be generated in sediment.

**Fig. 3:**
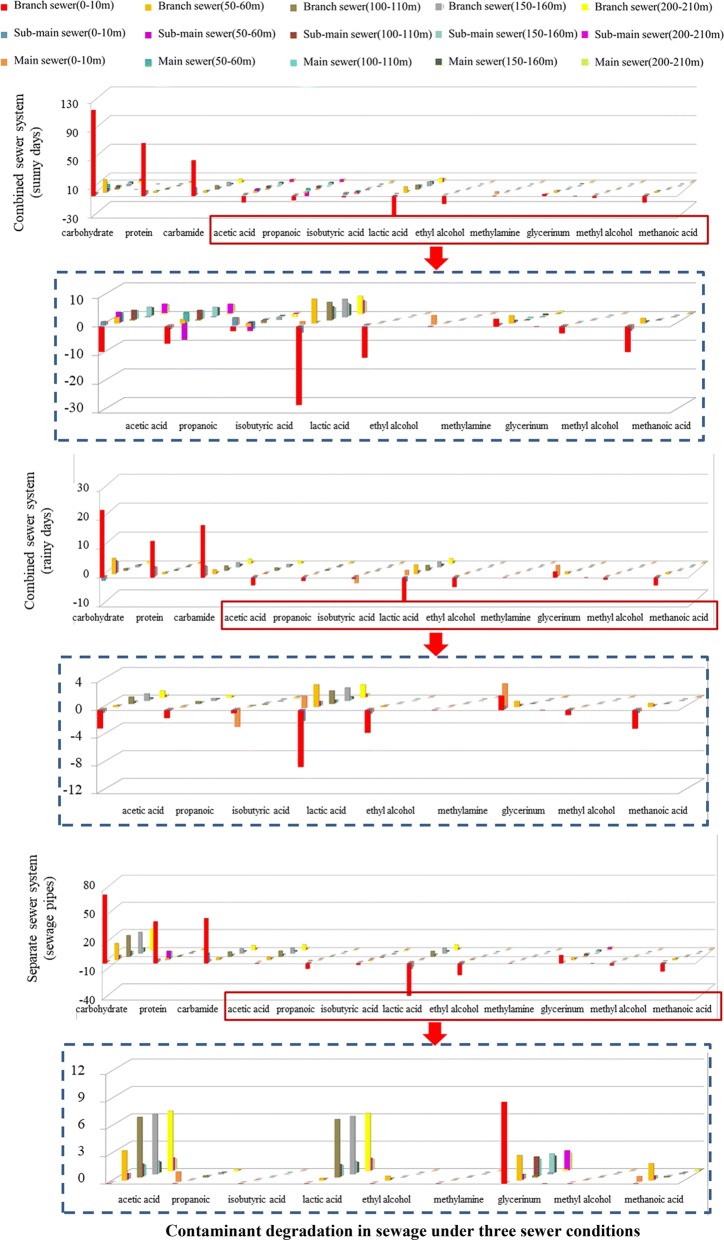
Degradation of diverse pollutants under three sewer conditions

**Fig. 4:**
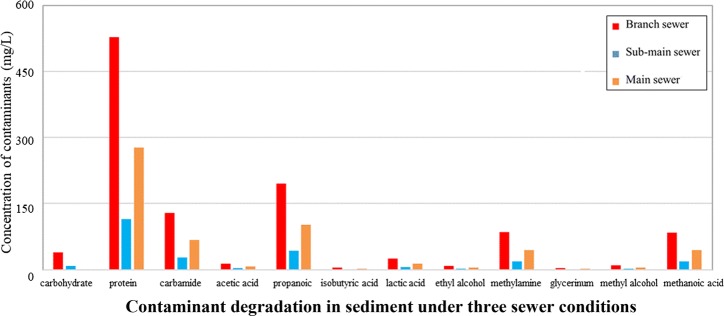
Degradation of diverse pollutants under three sewer conditions

According to the metagenomics analysis mentioned above, the macromolecule contaminants of carbohydrates, proteins and lipids, micromolecule contaminants of methyl alcohol, methanoic acid, methylamine, and so on were the node sources for CO_2_ and methane generation, therefore, those contaminants were monitored for 3 years along branch sewer, sub-main sewer, and main sewer, respectively. The average monitoring results can represent full range of greenhouse gas emission under the effect of weather condition and different systems. As shown in Fig. 3, the concentrations of methylamine, methanol, formate, and acetate in sewage decreased and that of methane increased along the real urban sewer system. In addition, the carbohydrates and proteins in sewage decomposed along the sewer system, and could provide abundant substrates for CO_2_ generation. The degradation of organic matter in the combined system was generally similar to that in the separate sewer system. The concentrations of polysaccharides, proteins, and lipids in branch pipes were significantly higher than those in main pipes (for 112 mg/L, 42 mg/L, and 6 mg/L, respectively), indicating that these organic materials in sewage were degraded along the sewer. Previous studies showed that sediment was ubiquitous along the urban sewer and was contributed by the deposition of particulate matter from sewage [8, 17]. To determine the pollutant degradation in sediment, 10 meters of sediment was taken from the sewer. As shown in Fig. 4, under the flow of sewage, the concentration of diverse pollutants such as macromolecular pollutants (carbohydrate, protein, and lactic acid) and micromolecular pollutants (acetic acid, methylamine, and methyl alcohol) decreased in sediment. The results also showed that the concentration of the degradative mass in sediment was highest in branch pipes. For example, the concentration of protein was 531 mg/L in the sediment of branch pipes, and it decreased rapidly to 122 mg/L in the sediment of sub-main pipes. Although the convergence phenomenon occurred in main pipes, the concentration of protein (276 mg/L) was still lower than that in branch pipes, which was resulted from the significant degradation of pollutants in upstream pipes. Based on the high-throughput analysis mentioned above (as shown in Fig. 2), functional microbial communities such as *Trichococcus*, *Cloacibacterium*, and *Paludibacter* were abundant in sewer sediment. Their abilities on hydrolysis and acidification of pollutants were the main reasons for the generation of CO_2_ and methane [18–20]. Thus, it indicated that the contributions to greenhouse gas generation by sewage and sediment changed in the different levels of sewer, and this characteristic should be considered in the calculations of greenhouse gas emission in sewers.

### Calculation of the greenhouse gas emission from sewer systems

**Fig. 5:**
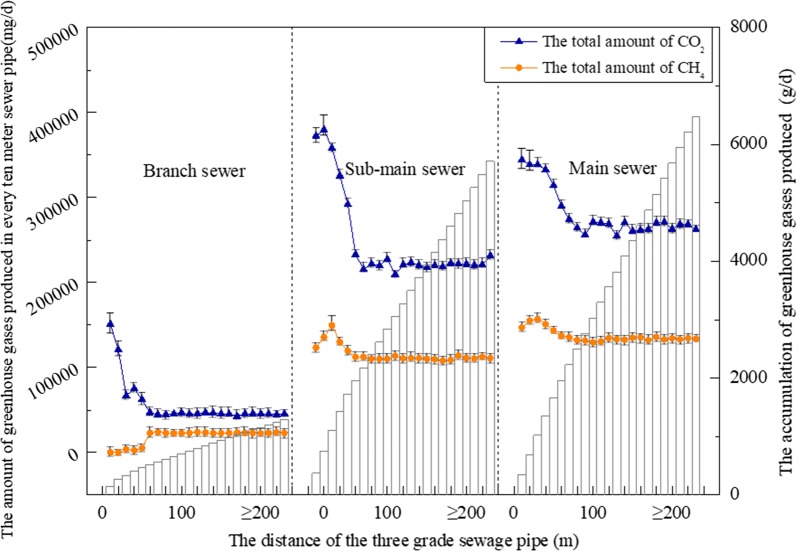
The total generation amount of CO_2_ and methane in sewer system

Based on the monitoring results of node contaminants in sewers, the generation values of CO_2_ and methane can be calculated through the equations which are obtained from the contaminant transformation venation (as shown in Additional file 5: Figure S5 and “Methods” section). As shown in Fig. 5 and Additional file 2: Figure S2a, there were four transformation pathways that utilized carbohydrates, three transformation pathways that utilized proteins and three transformation pathways that utilized lipids during the CO_2_ generation process in sewer. All the transformation pathways induced the continuous accumulation of CO_2_ in the branch sewer (4.6 × 10^4^ mg/day), sub-main sewer (2.2 × 10^5^ mg/day), and main sewer (2.7 × 10^5^ mg/day), therefore, the accumulation rates of CO_2_ gradually increased in the three levels of sewer, and the highest accumulation rate was found in the main sewer. It should be noted that at the confluence segment of the three levels of sewer, the generation rate of CO_2_ reached to a peak value and then maintained a steady state along the sewer. This phenomenon was especially significant at the beginning segment of branch sewer and might have been due to the high concentrations of carbohydrates, proteins, and lipids present in this segment, which provided abundant substrates for the generation of CO_2_. The substrates were consumed by the microbial communities in the rear segment. Therefore, the generation rate of CO_2_ gradually achieved a steady state. As shown in Fig. 5 and Additional file 2: Figure S2b, there were four transformation pathways (utilizing methyl alcohol, methanoic acid, methylamine, and acetic acid) for methane generation in sewer which induced the continuous accumulation of methane in the branch sewer, sub-main sewer, and main sewer. The highest generation rate of methane occurred in the main sewer (1.3 × 10^5^ mg/day), and this was also observed for CO_2_ generation. In addition, the emission rates of greenhouse gas in sediment were higher than those in sewage in branch pipes. They were basically the same as those in sewage in sub-main pipes, but were much lower than those in sewage in main pipes. Due to the smaller pipe diameter of branch pipes, sediment formed easily in them, and the abundant pollutants provided a suitable environment for the generation of greenhouse gases. With the increased flow velocities and larger pipe diameters of the sub-main and main pipes, the sediment mass was gradually decreased. Therefore, the generation rates of greenhouse gas were lower than in sewage.

The total lengths of the three levels of urban sewer network in Xi’an were 247,521 m, 219,506 m, and 371,873 m, respectively. Therefore, the CO_2_ production in branch pipe, sub-main pipe, and main pipe in Xi’an was calculated to be 1.13 t/day, 4.85 t/day, and 10.12 t/day, respectively, and the methane production was 0.57 t/day, 2.43 t/day, and 4.96 t/day, respectively. The conversion indexes of CO_2_ and methane to greenhouse gas are 1 and 23, respectively [16], so the total greenhouse gas emissions in sewer was calculated to be 199 t/day, which was basically the same as the monitoring results. It indicated that the calculation methods were reasonable.

### Effect of greenhouse gas emission from urban sewer systems

**Fig. 6:**
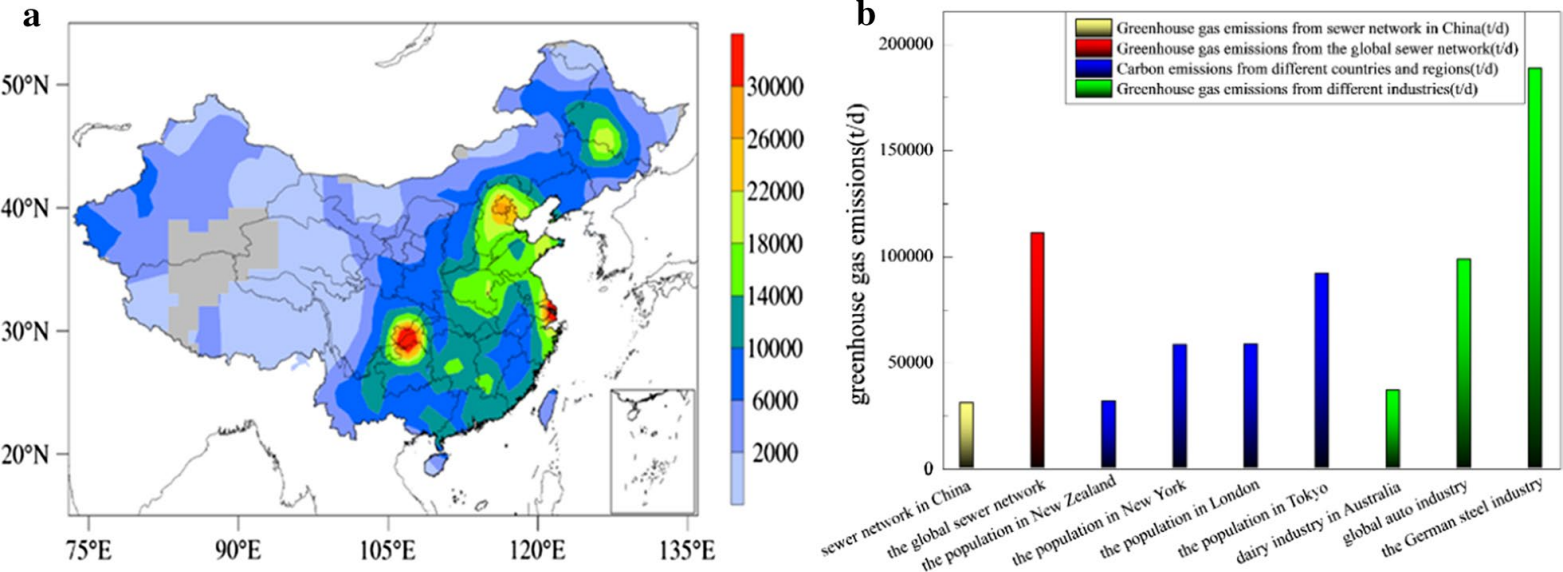
**a** Distribution of greenhouse gas emission from urban sewer systems in China and **b** amounts of greenhouse gas emission from industries in the world

As mentioned above, the total greenhouse gas emission amount from the urban sewer network in Xi’an is 199 t/day. The total population of Xi’an is 8,705,600, and the total population of China is equivalent to 154 times that of Xi’an. Therefore, it can be estimated that the greenhouse gas emission from sewer systems in China is 30,685 t/day. Based on the characteristics of population distribution in China, the greenhouse gas emission amounts differ in different regions of China. As shown in Fig. 6a, the greenhouse gas emission amounts in Sichuan Basin (southwest of China) and eastern coastal cities are extremely high, but are comparatively low in the western regions. In addition, the per capita carbon footprint in Xi’an is approximately 2.483 t/year [21], which is 6.803 × 10^−3^ t/day. Based on this index, the total amount of greenhouse gas emission from the sewer systems in Xi’an is equivalent to the emissions from 29,300 people, and the amount of greenhouse gas emission in China is equivalent to the emissions from 4.51 million people which is basically equal to the entire population of New Zealand. As shown in Fig. 6b, the amount of greenhouse gas emission from urban sewers in China is close to the emission from the total population of New Zealand. In addition, the greenhouse gas emissions from the total population of New York City, London, and Tokyo can be calculated to be 58,171 t/day, 58,512 t/day, and 91,897 t/day, respectively.

Based on the concept of life cycle, Flysjö et al. [22] established a carbon footprint assessment framework and related standards for dairy farms, and used this method to calculate the carbon footprint for the production of 1 kg standard milk (FPCM) (1.34 kgCO_2_). The amount of milk produced in Australia in 2016 was 10 million tons, so the amount of greenhouse gas emissions can be calculated to be 36,749 t/day. Lee [23] established models to evaluate carbon emission and found a value of 379 kg/car for the four major manufacturing processes and material movements in the automobile production system; and the global car output in 2016 was 95 million, which led to the release of 98,585 t/day of greenhouse gases. Li [24] established a model for calculating carbon dioxide emissions from iron and steel enterprises and summarized the carbon dioxide emission from each process in Baosteel. The carbon emissions during this process are 1636 kg/t produced, and annual output of iron and steel is 42 million tons in Germany in 2016, so the total amount of greenhouse gas emissions is 188,691 t/day. The amount of greenhouse gases produced by the global urban sewer system is much higher than the amounts produced by the above industries, which indicates that the amount of greenhouse gas emitted from the urban sewer systems should not be underestimated and need much more attention. In addition, raising feasible strategies to mitigate the greenhouse gas emissions coming from sewer systems should be considered. Previous studies have demonstrated that air/pure oxygen injection could be adopted to mitigate sulfide generation in sewer systems [10, 25]. The formation of greenhouse gases occurred under the anaerobic environment which was the same as sulfate-reducing process, therefore, changing the condition of dissolved oxygen could also be considered to mitigate greenhouse gas emissions. Furthermore, the greenhouse gas generations were contributed by the metabolism of functional microbial pathways, which have been illustrated in this study. If the biocides such as biocide-free nitrous acid (FNA) are added in sewers, the biophase dynamics can be prohibited, and those methods were also proposed by many scholars to mitigate sulfide generation [26, 27]. However, changing the environment and adding the biocides in sewers are uneconomical and cannot guarantee long-term effect, therefore, the new feasible strategies to mitigate the greenhouse gas emissions should be continually explored.

## Conclusion

In this study, contaminant degradation and greenhouse gas emission were investigated in the urban sewer system at Xi’an, China. The results showed that the generation processes of CO_2_ and CH_4_ were basically the same as the results from this investigation along the branch pipes, sub-main pipes, and main pipes. In addition, based on the carbon footprint results, the total amount of greenhouse gas emissions from sewers was compared with the emissions from important industries such as milk production, automobiles, and steelworks. These results showed that the greenhouse effect of urban sewers was much more serious than those of these industries. Thus, more attention should be paid to the greenhouse gas emissions from sewers and great efforts need to be made to improve the operation of sewer systems to reduce carbon emissions.

## Methods

### The experimental procedures in this study

**Fig. 7:**
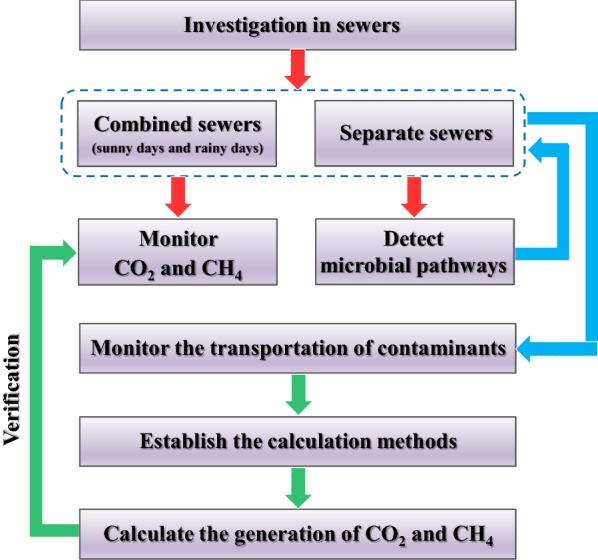
Flowchart of the experimental procedure (Red arrows represent procedure 1; blue arrows represent procedure 2; green arrows represent procedure 3)

This study was conducted in eight typical areas of Xi’an, China. As shown in Additional file 3: Figure S3, the selected areas include new urban districts (at the southeast and north parts in the map) and old towns (at the central and eastern parts in the map) where residential areas, comprehensive service areas, schools, and business districts are located (as shown in Additional file 4: Figure S4). The separate sewers mainly exist in new urban districts and the combined sewers mainly exist in old towns. Overall, 37 km of sewer systems was monitored over 3 years and covered 13km^2^. The selected sewer systems include 52 segments of main sewer, 84 segments of sub-main sewer, and 129 segments of branch sewer. Both the contaminants in the sewage and the greenhouse gases were monitored in inspection wells of the sewers. In order to reveal the effect of weather conditions on contaminant transportation in sewers, the samples were collected in sunny days and rainy days. All of the samples were collected along the target pipes at the same time. As shown in Fig. 7, the detailed experiment procedures are as follows: firstly, the CO_2_ and methane concentration along the sewers were monitored, and the biofilms and sediments were sampled to detect the microbial pathways; after that, the variation of contaminants was monitored according to the results of bioanalysis, and then, the calculation methods of CO_2_ and methane were established to analyze the process of greenhouse gas generation; in the end, the calculation results were validated with the monitoring results to guarantee the correctness.

### Calculation methods for greenhouse gas generation in all pathways

During sewage transportation in urban sewer systems, the many microbial communities that are present consume the substrates in the sewage to generate greenhouse gases. In this study, the transformation of organic matter and the dominant bacteria in the sewer system were analyzed, and the amounts of greenhouse gases produced by various biochemical reactions in different sewer segments were calculated. Urban sewer systems are mostly under anaerobic conditions. Therefore, contaminants in the sewer are primarily degraded by anaerobic biochemical reactions. According to the three-step theory of anaerobic fermentation [28], the microbial communities participating in the anaerobic biochemical reaction are represented by fermentative, hydrogen-producing acetogenic and methanogenesis communities, and these steps are accompanied by the generation of CO_2_ and CH_4_. In the fermentation stage, complex macromolecular organic substances are decomposed into small molecular organic matter, and particulate matter is decomposed into dissolved material [29]. For instance, protein can be decomposed into amino acids, polysaccharides can be decomposed into glucose, and lipids can be decomposed into fatty acids and glycerol [30]. The generation of these small molecular organic compounds in the first step can induce the emission of CO_2_ [31]. Then, in the hydrogen-producing acetogenic step, small molecules such as propionic acid, isobutyric acid, and lactic acid can be consumed by fermentative bacteria to generate CO_2_. Finally, methanogens can consume formic acid, methanol, methylamine, and acetic acid to produce methane in the third step. H_2_ is also an important substrate that can be used in methane production. The specific transformation pathways of CO_2_ and CH_4_ in the sewer are shown in Additional file 5: Figure S5.

For example, in the case of glucose degraded to butyric acid and CO_2_, the formula for calculating CO_2_ formation is as follows:

1 $$\Delta n_{{{\text{CO}}_{ 2} }} = \frac{{b_{{{\text{CO}}_{2} }} }}{{b_{{{\text{C}}_{4} {\text{H}}_{8} {\text{O}}_{2} }} }} \cdot \Delta n_{{{\text{C}}_{4} {\text{H}}_{8} {\text{O}}_{2} }} = \frac{{b_{{{\text{CO}}_{2} }} }}{{b_{{{\text{C}}_{4} {\text{H}}_{8} {\text{O}}_{2} }} }} \cdot \frac{{\left( {C_{{A_{1} }} - C_{{A_{0} }} } \right) \cdot V}}{{M_{{{\text{C}}_{4} {\text{H}}_{8} {\text{O}}_{2} }} }}$$

2 $$\Delta n_{{{\text{C}}_{4} {\text{H}}_{8} {\text{O}}_{2} }} = \frac{{m_{{{\text{C}}_{4} {\text{H}}_{8} {\text{O}}_{2} }} }}{{M_{{{\text{C}}_{4} {\text{H}}_{8} {\text{O}}_{2} }} }} = \frac{{\left( {C_{{A_{1} }} - C_{{A_{0} }} } \right) \cdot V}}{{M_{{{\text{C}}_{4} {\text{H}}_{8} {\text{O}}_{2} }} }}$$

3 $$\Delta m_{{{\text{CO}}_{ 2} }} = \Delta n_{{{\text{CO}}_{ 2} }} \cdot M_{{{\text{CO}}_{ 2} }}$$

4 $$\Delta m_{{{\text{CO}}_{ 2} }} = \frac{{b_{{{\text{CO}}_{2} }} }}{{b_{{{\text{C}}_{4} {\text{H}}_{8} {\text{O}}_{2} }} }} \cdot \frac{{\left( {C_{{A_{1} }} - C_{{A_{0} }} } \right) \cdot V}}{{M_{{{\text{C}}_{4} {\text{H}}_{8} {\text{O}}_{2} }} }} \cdot M_{{{\text{CO}}_{ 2} }},$$

where *V* is the tube segment volume (m^3^), $C_{{A_{0} }}$ and $C_{{A_{1} }}$ are the butyrate concentration in the pipe segment of *A*_0_ and *A*_1_, $M_{{{\text{CO}}_{2} }}$ and $M_{{{\text{C}}_{4} {\text{H}}_{8} {\text{O}}_{2} }}$ are the molar masses of CO_2_ and C_4_H_8_O_2_ (g/mol), $m_{{{\text{CO}}_{2} }}$ and $m_{{{\text{C}}_{4} {\text{H}}_{8} {\text{O}}_{2} }}$ are the masses of CO_2_ and C_4_H_8_O_2_ (g), $n_{{{\text{CO}}_{2} }}$ and $n_{{{\text{C}}_{4} {\text{H}}_{8} {\text{O}}_{2} }}$ are the amount of CO_2_ and C_4_H_8_O_2_ (mol), $b_{{{\text{CO}}_{2} }}$ and $b_{{{\text{C}}_{4} {\text{H}}_{8} {\text{O}}_{2} }}$ are the stoichiometric number of CO_2_ and C_4_H_8_O_2_.

Thus, the general formula can be obtained as follows:

5 $$\Delta m_{{{\text{CO}}_{ 2} }} = \frac{{b_{{{\text{CO}}_{2} }} }}{{b_{x} }} \cdot \frac{{\left( {C - C_{0} } \right) \cdot V}}{{M_{x} }} \cdot M_{{{\text{CO}}_{ 2} }} ;\Delta m_{{{\text{CH}}_{ 4} }} = \frac{{b_{{{\text{CH}}_{ 4} }} }}{{b_{x} }} \cdot \frac{{\left( {C - C_{0} } \right) \cdot V}}{{M_{x} }} \cdot M_{{{\text{CH}}_{ 4} }}$$

This method can also be used to calculate the degradation pathways of carbohydrates, proteins, and lipids that generate CO_2_ and CH_4_ in urban sewers.

### Chemical analysis

#### Analysis of VFAs

Phosphoric acid (3%) was dropwise added to regulate the pH of the solution to approximately 4, and the water sample was filtered with a 0.45-μm filter membrane. The hydrogen flame ionization detector (FID) was used in a GC-2014 gas chromatograph (SHIMADZU, Japan). A DB-FFAP-123-3233 capillary column (30 m × 0.5 μm × 0.32 mm), with a sample size of 1 μL per sampling, was adopted to detect the SCFAs. The temperature of the sample injection port was set to 200 °C, and the detector temperature was set to 230 °C. The procedures used to increase the temperature of the instrument (oven of GC) were as follows: the column temperature was initially maintained at 100 °C for 2 min; the temperature was then increased to 120 °C at a rate of 10 °C/min and later kept at 120 °C for 2 min; the temperature was increased to 200 °C at a speed of 6 °C/min and held at 200 °C for 2 min. The total elapsed time of the temperature-increasing protocol was 21.33 min.

#### Analysis of methanol

The analytical instrument was a GC-2014 gas chromatograph (SHIMADZU, Japan) equipped with an AOC-5000 headspace automatic sampler and the chromatographic column was CB-5 (30 m × 0.25 μm × 0.32 mm). The procedures used to increase the temperature of the instrument (oven of GC) were as follows: the column temperature was initially maintained at 50 °C for 5 min; the temperature was increased to 100 °C at a rate of 5 °C/min and later kept at 100 °C for 2 min; and the temperature was increased to 200 °C at a speed of 5 °C/min and held at 200 °C for 2 min. The total elapsed time of the temperature-increasing protocol was 39 min.

#### Analysis of formic acid

A LC2010AHT liquid chromatograph (SHIMADZU, Japan) was used to analyze the formic acid. The chromatographic column was Hypersil BDS C18 (250 mm × 4.6 mm × 5 m), and the UV detector was set at 210 nm. The mobile phase was 0.02 mol/LKH_2_PO_4_ methanol with a volume ratio of 95:5, and the phosphoric acid was used to adjust the pH to approximately 2. The flow rate was set to 1.0 mL/min, and the column temperature was 30 °C.

#### Analysis of methylamine

Before detection, samples were treated by PITC derivatization, and then solid phase extraction was carried out with C18 columns. The LC2010AHT liquid chromatograph (SHIMADZU, Japan) was used. The chromatographic column was a Hypersil BDS C18 (250 mm × 4.6 mm × 5 m), and the UV detector was set at 240 nm. The flow phase was acetonitrile water with a volume ratio of 30:70. The flow rate was set to 1.0 mL/min, and the column temperature was 30 °C.

#### Analysis of CH_4_ and CO_2_

The medical blood vessel was used to collect the gas samples (CH_4_ and CO_2_). Gas chromatography (Agilent 6890 gas chromatograph) was used to measure the concentrations of CH_4_ and CO_2_. A thermal conductivity detector (TCD) and a TDX-01 filled column were used. The column temperature was set at 100 °C, and maintained for 10 min. The standard gas mixture was calibrated by 37% CO_2_, 4% N_2_, 0.802% H_2_, and CH_4_.

#### High-throughput sequencing and metagenome analysis

Illumina Miseq sequencing, 454 pyrosequencing, and metagenome analysis were performed to analyze the distribution of the microbial community and the annotated pathways in this study. Sequencing was conducted by the Novogene Institute (Beijing, China). The total genomic DNA was extracted from the samples using the CTAB/SDS method. The DNA concentration and purity were monitored on 1% agarose gels. According to the concentration, DNA was diluted to 1 ng/μL using sterile water.

## Additional files

**Additional file 1: Figure S1.** The types of water quality in four functional areas in sewer systems.

**Additional file 2: Figure S2.** The generation process of CO_2_ and methane along the sewer system.

**Additional file 3: Figure S3.** A sketch map of the selected research sites in Xi’an.

**Additional file 4: Figure S4.** Schematic diagram of different sewer systems in eight functional areas.

**Additional file 5: Figure S5.** The greenhouse gas generation pathways in urban sewer system.
