# Supplementary figures and images for: Non-negligible greenhouse gases from urban sewer system
Source: Biotechnol Biofuels. 2019 Apr 27;12:100. doi: 10.1186/s13068-019-1441-8 (PMC6486696; doi:10.1186/s13068-019-1441-8)

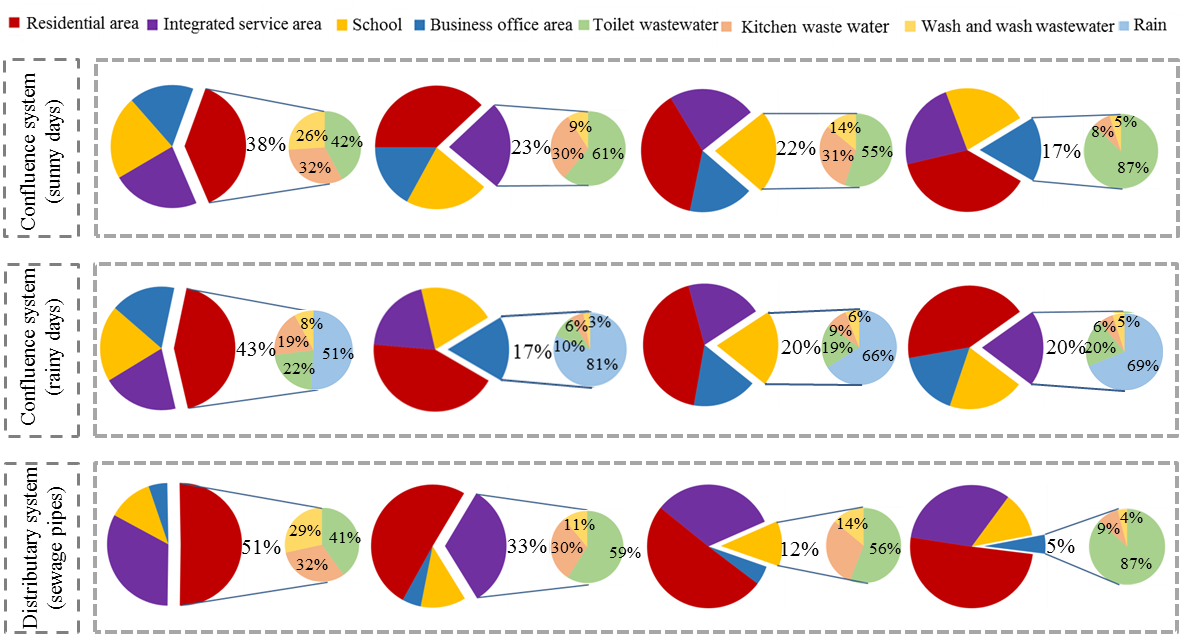


Figure S1 The types of water quality in four functional areas in sewer systems

Supplement: Supplementary file 1 — Additional file 1: Figure S1. The types of water quality in four functional areas in sewer systems. [file 13068_2019_1441_MOESM1_ESM.doc]

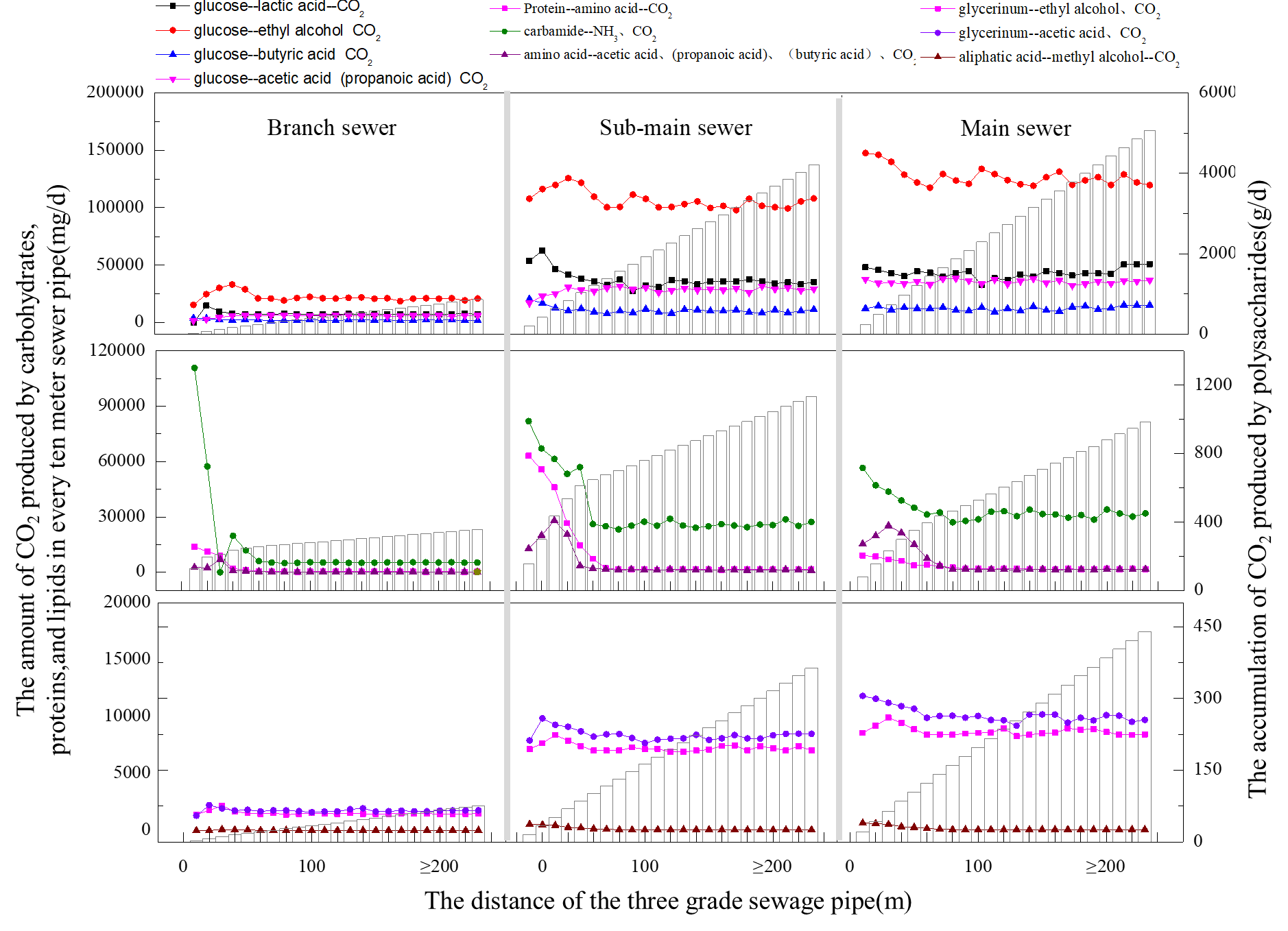


(a)


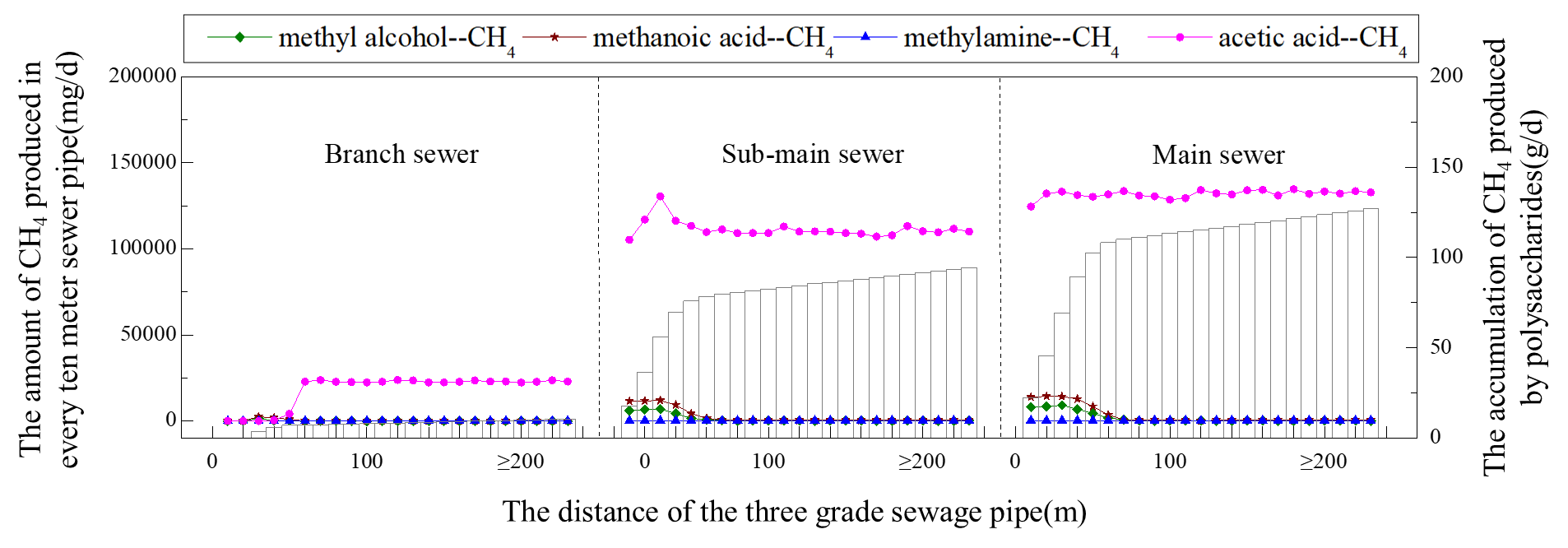


(b)

Figure S2 The generation process of CO2 and methane along the sewer system

Supplement: Supplementary file 2 — Additional file 2: Figure S2. The generation process of CO2 and methane along the sewer system. [file 13068_2019_1441_MOESM2_ESM.doc]

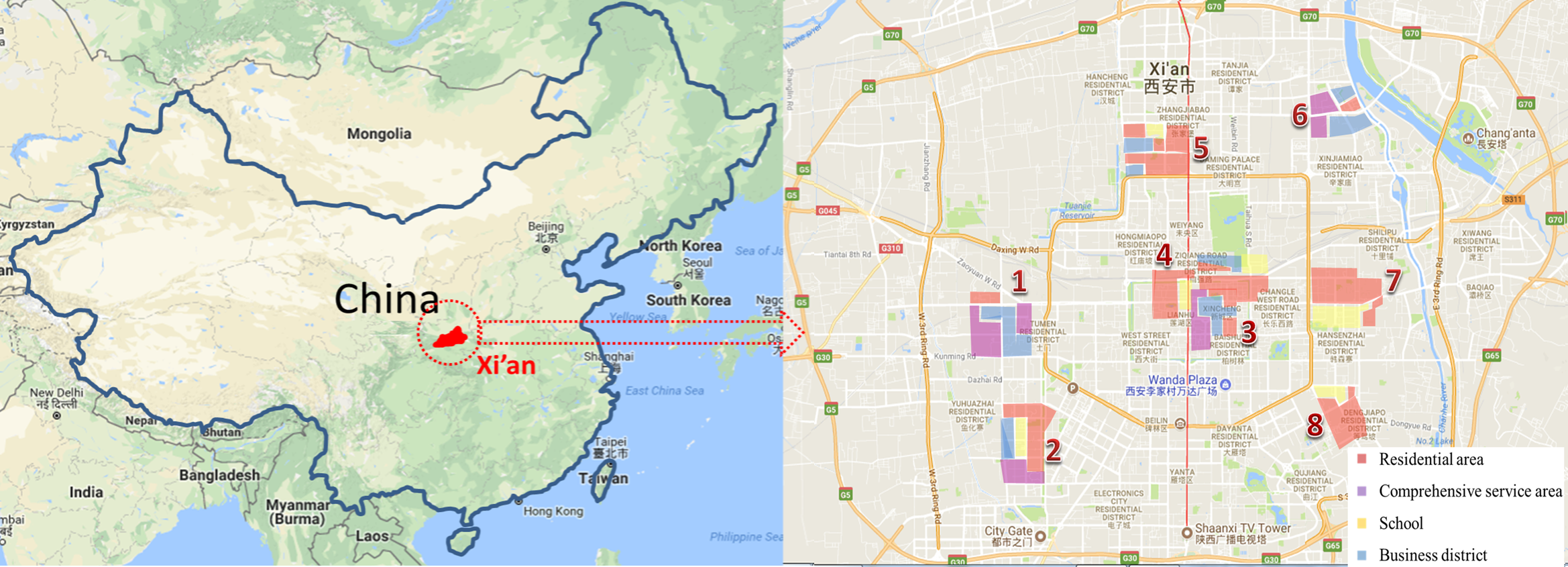


Figure S3 A sketch map of the selected research sites in Xi'an

Supplement: Supplementary file 3 — Additional file 3: Figure S3. A sketch map of the selected research sites in Xi’an. [file 13068_2019_1441_MOESM3_ESM.doc]
