# Supplementary material for: Non-negligible greenhouse gases from urban sewer system
Source: Biotechnol Biofuels. 2019 Apr 27;12:100. doi: 10.1186/s13068-019-1441-8 (PMC6486696; doi:10.1186/s13068-019-1441-8)
Supplement: Supplementary file 4 — Additional file 4: Figure S4. Schematic diagram of different sewer systems in eight functional areas. [file 13068_2019_1441_MOESM4_ESM.doc]

Figure S4 Schematic diagram of different sewer systems in eight functional areas
